# Comparison of Corrosion Resistance of Cu and Cu72Zn28 Metals in Apricot Fermentation Liquid

**DOI:** 10.3390/ma18061253

**Published:** 2025-03-12

**Authors:** Stevan P. Dimitrijević, Silvana B. Dimitrijević, Andrea Koerdt, Aleksandra Ivanović, Jelena Stefanović, Tanja Stanković, Husnu Gerengi

**Affiliations:** 1 Innovation Centre, Faculty of Technology and Metallurgy, University of Belgrade, Karnegijeva 4, 11120 Belgrade, Serbia; 2 Mining and Metallurgy Institute Bor, Alberta Ajnštajna 1, 19210 Bor, Serbia; silvana.dimitrijevic@irmbor.co.rs (S.B.D.); aleksandra.ivanovic@irmbor.co.rs (A.I.); jelena.stankovic@irmbor.co.rs (J.S.); tanja.stankovic@irmbor.co.rs (T.S.); 3 Bundesanstalt für Materialforschung und Prüfung (BAM), Unter den Eichen 87, 12205 Berlin, Germany; andrea.koerdt@bam.de; 4 Corrosion Research Laboratory, Department of Mechanical Engineering, Faculty of Engineering, Duzce University, Duzce 81620, Türkiye; husnugerengi@duzce.edu.tr

**Keywords:** apricot fermentation, MIC, *Saccharomyces cerevisiae*, corrosion behavior, Cu72Zn28 alloy

## Abstract

The production of fruit brandies is based on distilling fermented fruit juices. Distillation equipment is usually made of copper. In traditional manufacturing, it consists of a boiler (batch) distiller, a boiler (pot), a steam pipe, and a condenser, all of which are made of pure copper. This study determined the corrosion parameters for copper (Cu) and Cu72Zn28 (in wt%) alloy in fermented apricot juice at room temperature. The fermentation process examined in this research utilized natural strains of yeast and bacteria, supplemented by active dry yeast *Saccharomyces cerevisiae* strains. This research used the following methods: open circuit potential (OCP), linear polarization resistance (LPR), and Tafel extrapolation to identify corrosion parameters. Cu had a 3.8-times-lower value of corrosion current density than brass, and both were within the range of 1–10 μA·cm^−2^, with an excellent agreement between LRP and Tafel. This study proved that Cu is an adequate material for the distillation of fruit brandies from a corrosion perspective. Despite this, there are occasional reports of corrosion damage from the field. Significant corrosion impacts can arise, as evidenced by laboratory tests discussed in this paper. In the absence of a highly corrosive environment, this study indicates that, to some extent, microbiologically influenced corrosion (MIC) can influence the degradation of the equipment material.

## 1. Introduction

Studies on the corrosion of materials used in specific food applications are not as common as those for standardized food industry applications. This is especially true for copper (Cu) and different types of brass. Brass corrosion in different media has been investigated [1], as well as the influence of various (standard) media on cooking utilities [2]. This study investigated the corrosion of Cu and brass (72Cu-28Zn, in wt%) in fermented apricot juice, an interesting corrosion environment consisting of about 6–7% ethanol, nearly 0.5% acetic acid, water, metals (K, Ca, Mg) in the range of mg/L to tens of mg/L, and yeast strains, with a slightly acidic pH (3–4). In contrast to bacteria, these organisms severely affect aluminum but not other materials (Cu, brass, and stainless steel) [3]. Stainless steel 304 has by far the best corrosion resistance of those materials in tequila (as expected), followed by brass and Cu with similar behavior (unexpectedly brass had a little lower corrosion current) and aluminum with about a ten-times-higher corrosion rate than Cu or brass [3]. However, this was not directly related to microbiologically influenced corrosion (MIC) because fermentation was finished, and the solution was distillate without living microorganisms.

The influence of zinc in brass on corrosion properties is significant and not consistently negative. Zinc affects the kinetics of corrosion parameters and could lower the anodic Tafel slope of Cu [4,5]. Due to dezincification, brass has a higher corrosion rate than Cu in neutral chloride media and alkaline solutions. In acid solutions, it is generally slightly less corrosion resistant, usually with a low margin [6,7]. However, it can have comparable or even slightly higher resistance in non-complex acidic media, such as 1M nitric acid [8,9] and a combination of 1 M sodium sulfate and 0.1 M sulfuric acid, where the value of the dezincification factor was nearly one (absence of the dealloying process) at higher electrode potentials [10]. The corrosion behavior of brass in drinking water systems is very important since it is the construction material for a valve that is used there, and it could be influenced by MIC [11].

Corrosion in acetic acid is the topic which is mainly addressed in this paper. So far, not many studies comparing Cu and brass have been performed, except numerous papers dealing with the protection effects of some organic acids on different metals and alloys, including Cu and brass, as additive systems in ethylene glycol–water coolants [12,13]. However, despite similarities, inhibition is quite a different subject. A direct comparison study found the weight-loss method nearly doubled the corrosion rate for brass in 4 M CH_3_COOH compared with Cu [6]. A corrosion study of Cu in 1% formic and acetic acid showed that corrosion current densities initially were low (about or under 1 μA·cm^−2^) but increased significantly with immersion time, and formic acid was more corrosive [14]. A recent study on brass (CuZn_36_Si_1_) corrosion in acetic and citric acids showed that citric acid was a less corrosive medium. The corrosion rate for acetic acid was in the range of 66 to 91 mg/m^2^·h for concentrations from 0.25 M to 2 M, with logarithmic dependence. The stirring of the solution and the presence of Cu^2+^ ions highly increased the corrosion rate [15].

Many organic complexes can serve as corrosion inhibitors [13,16,17]. Several investigations have been performed to study the inhibitory effect of plant extracts on corrosion [18,19,20]. However, this complicates studying complex organic solutions with microorganisms, organic acids, flavonoids, and other ingredients. In addition to good corrosion resistance characteristics, alpha brasses (especially those with a Cu content ≥70 wt%) also have excellent thermal properties (high thermal conductivity of over 100 W/mK, about a third of that of Cu) and do not deteriorate much with exposure to corrosive environments [7,17]. Due to these features, alpha brasses have found applications similar to those in this manuscript, such as coolers and heat exchangers, in various industries [21]. Further, brass has found application in novel, innovative equipment like modular heat exchangers with metal foam packing [22] and brass wire mesh blocks [23].

However, it is often related to the failure of the cooling (heat exchanging) equipment and failure assessments in industrial plants which is frequently connected with corrosion problems [24], especially dezincification [25,26,27]. Although Cu and brass are materials considered to have good resistance to MIC [27], some research has found that they are sensitive to it [28]. One study revealed the influence of calcium-depositing bacteria on the corrosion of several engineering metals, including Cu and brass [29]. It showed that brass had a significantly higher corrosion rate (weight loss method) than Cu and about a four-fold higher corrosion current density, as determined by Tafel polarization. A relevant negative role on passivation by biofouling deposition on CuNi30 and Al brass condenser tubes was confirmed in a study [30] and microbial-assisted cracking of admiralty brass tubes in another [31]. However, the positive influence of microorganisms by the inhibition of food processing brass dezincification in the presence of *Pseudomonas fluorescens* was documented in a publication [32]; it was found that bacteria decrease oxygen content.

Although it sounds paradoxical, the following sentence summarizes the controversy excellently [33]: “Copper and its alloys are vulnerable to MIC despite their generally good corrosion resistance and antibacterial properties”. The metabolic actions of microorganisms within biofilm can lead to higher oxygen concentrations, different pHs, and changes of the concentration of different species, including organics, than in the solution. Microbial products of metabolism, including CO_2_ and organic acids, may cause the MIC of Cu or its alloys [34]. The dual role of oxidized copper (primarily Cu_2_O) has contributed to controversial results in different studies. However, certain studies have fairly clarified this. Copper oxides (Cu_2_O and CuO) are less effective antibacterial agents than pure Cu metal. Still, they can be beneficial for alloys with lower Cu content [35,36] or even make a surface film with a high MIC resistance [36]. The current research obtained Cu_2_O as the main corrosion product but has shown that, although uniform, it was porous with little influence as an inhibition (protection) film.

Metabolites, especially acidic (CO_2_ and volatile fatty acids), increase the corrosion rate of Cu alloys by an opposite effect of inhibition due to the influence of living microorganisms [37]. This presents a significant issue for the MIC impact on the Cu equipment in contact with microbial metabolites, as is the case for the distilleries. Corrosion inevitably leads to Cu release in solution or pulp that will be subjected to the distillation. A high Cu concentration can influence the fermentation process, and it was reported that 0.39 mmol of Cu concentration decreased the vitality of yeast (*Saccharomyces cerevisiae*) in must [38]. Still, it is much higher than the permitted limit for commercial products in Brazilian (5.00 mg/L) or EU (2.00 mg/L) legislation [39]. Higher than 2 mg/L of Cu was detected in about 40% of 33 distilleries in the published study, and 15.15% with ≥5.0 mg/L [40]. Although it was focused on ethyl carbamate in spirits, proposed measures for improvement (reduce level) are the same for both, and the most important was to minimize exposure to copper (by using stainless steel) in some parts of the distillery equipment, although not including the pot still.

The properties of alpha brasses and the earlier discussed results in published papers establish them as an appropriate control for comparison with Cu, which was utilized in this paper. This study presents research results about Cu as a construction material for the distillation process and brass as a control in fermented fruit juice. The main subject of this study was to establish and compare the main corrosion parameters for Cu and its Cu72Zn28 alloy in the product of apricot pulp fermentation in partially controlled but fully measured parameters in the real system. The lack of details is sacrificed to authenticate the production conditions on the rural farm. Although specific, this is a real-world scenario (taken from actual production) in fruit brandy distillation. With all its restrictions and simplifications, the scenario from this study represents a good base for the industrial issues in the same area. The novelty and scientific significance of this paper are that it copes with the controversial theme, with different results in the literature, of Cu and Cu alloy’s susceptibility to MIC, which is here studied on the level of microbial metabolic products.

## 2. Materials and Methods

Experimental research was carried out in fermented apricot juice on Cu and a-brass electrodes embedded in polyurethane resin. E-pH diagrams were constructed using the HSC Chemistry version 6.12 software package (Outotec Research Oy, Pori, Finland). Electrodes were made from Cu with 99.98 wt% purity (nominally ≥99.97 wt%); CuZn alloy had the composition: 71.63 wt% Cu, 28.27 wt% Zn (XRF, Niton XL3t-900, Thermo Fisher Scientific, Waltham, MA, USA; also used for Cu analysis), with total impurities lower than 0.05% (500 ppm)—determined using inductively coupled plasma optical emission spectroscopy (ICP-OES), Spectro Analytical Instruments GmbH (Kleve, Germany), Model: Ciros Vision, Detection limit: from ≤0.1 mg/L to ≤0.5 mg/L depending on metal, e.g., 0.2 mg/L for Ag).

Oenology active dry yeast in the form of pellets (product of Danstar Ferment AG, Fredericia, Denmark) containing *Saccharomyces cerevisiae* and E491 additive (sorbitan monostearate) was used as a fermenting agent. By specification, it contains >10^10^ CFU/g of viable yeast, more than 92% dry matter, lactic acid bacteria < 10^5^ CFU/g, acetic bacteria < 10^4^ CFU/g, molds < 10^3^ CFU/g, and yeast of different species < 10^5^ CFU/g. It was dosed in the concentration of 25 g/hl. Before addition to the must, the dry yeast was rehydrated as per the instructions of the producer (at a temperature of 37 ± 2 °C). Due to the traditional method of producing apricot brandy, the fermentation was not strictly controlled. Nevertheless, basic parameters were measured and kept within the set limits. Fermentation was performed in closed drums from polypropylene (PP), with the vent (and hose poured in water) for the realized CO_2_ to provide anaerobic conditions. The vessels were stored in the basement with a nearly constant temperature of 20 ± 2 °C during the process (mid-August to the end of the month). The must’s temperature was kept between 18 and 24 °C. Viscosity was slightly corrected with an initial addition of about 5% of dwell water. The pH value was kept between 4.3 (start) and 3.5 (end) and was mostly about 3.6–3.9 during the process. At the beginning of the process, a minor correction of pH was made by adding p.a. sulfuric acid (75 mL/hl). The fermentation lasted 9 days until the sugar content dropped below 4 °Bx (1 °Bx corresponds to 1 g of sucrose in 100 g of solution), which was controlled by a hand-held optical refractometer. An additional day was applied to the natural sedimentation of the yeast before the destination process.

**Figure 1:**
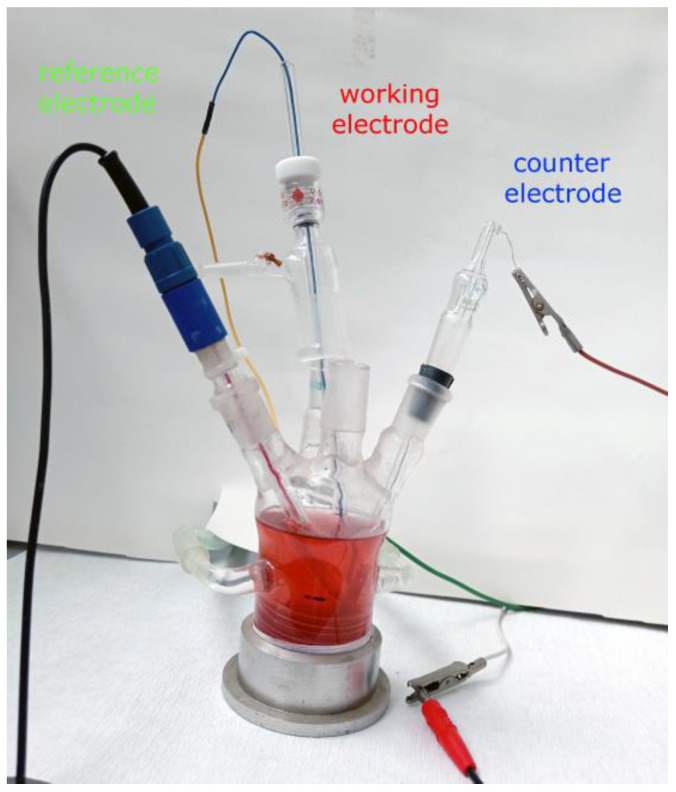
The electrochemical cell (system) with fermented apricot juice as the electrolyte (corrosion environment).

The instrument and the power source (potentiostat/galvanostat and Zero Resistance Ammeter) for electrochemical experiments were combined in one piece of equipment, the Gamry Interface 1000™ (Gamry Instruments Inc., Warminster, PA, USA). The software packages Gamry Framework and Echem Analyst (Version 6.25—both; Gamry Instruments, Warminster, PA, USA) were used for controlling electrochemical experiments, acquiring data, and analyzing experimental data. The electrochemical system in which the experiments were performed consists of an electrochemical cell with three electrodes, which is shown in Figure 1. Three of these electrodes were: saturated calomel electrode (SCE) as a reference, Cu and brass electrodes (area of 1 cm^2^, 2 mm of thickness) as working, and Pt sheet, with an area of 3 cm^2^, as a counter. The open circuit potential (OCP) experiment lasted 3600 s (one hour). The linear polarization resistance (LPR) and Tafel extrapolation methods were performed at a scan rate of 10 mV/min. The LPR was performed in the potential limits of ±20 mV from the OCP value, and the Tafel polarization curves were recorded in the range of OCP ± 200 mV. LSV parameters are given further in the text. Preparation of electrode surfaces was performed on Buehler (Buchler GmbH, Braunschweig, Germay) EcoMet 30 Auto/Manual Grinder & Polisher; firstly, they were ground by the use of SiC emery papers from micro grit (ANSI) grade 400 up to 1200, followed by polishing with 1 μm diamond paste. Between phases, and at the end, the samples were washed with double distilled water and finally degreased with petrol ether. The system was thermostated at 25 ± 1 °C.

The anodic films after LSV were characterized by X-ray diffraction, optical and scanning electron microscopy, and energy-dispersive X-ray spectroscopy (EDS). The X-ray diffraction patterns of tested samples were obtained on an EXPLORER: GNR (Analytical Instruments Group, Novara, Italy) diffractometer, operated at 40 kV and 30 mA, using Cu-K_α1/2_ radiation, wavelength 0.154 nm. The samples were 2 mm thick, which allows for compatibility with the instrument’s used holder. No additional treatments were applied to both original and oxidized samples of Cu and Cu72Zn28 metal. The morphology of corrosion films was studied using a scanning electron microscope (SEM) (JOEL Ltd. (Tokyo, Japan), model: JSM-IT300LV operated at 20 keV). The EDS spectra were recorded using an X-ray spectrometer (Oxford Instruments, Abingdon, UK). The anodized oxidized samples after OM and XRD were coated with gold (15 nm thick) using a JEOL JFC-1300 Auto Fine Coater (JOEL, Tokyo, Japan) for SEM-EDS analysis. Optical microscopy (OM) was performed using a Leica DM2700 M optical microscope (Leica Microsystems, Wetzlar, Germany). Leica’s customizable software (LAS V4.13) was used to research the structure of corrosion film. Reflected light type OM without preparation of samples was used in this study. The pH meter used to measure the acidity of the fermented juice was pH 3310 (WTW, Troistedt, Germany).

## 3. Results and Discussion

In this study, a detailed corrosion characterization of Cu and brass—Cu72Zn28 was performed. Corrosion parameters were determined by the use of electrochemical (EH) methods (linear polarization resistance and Tafel extrapolation). After these measurements, linear sweep voltammetry (LSV) was applied to both samples, with a scan speed of 10 mV/s, in the range from −0.8 V to +1.0 V for the brass sample and −0.7 to +1.1 V for the Cu electrode. A distinction in the potential ranges was made to bias a difference between the OCPs of these two electrodes. Following LSV treatment, samples were further investigated using SEM, OM, and XRD methods.

**Figure 2:**
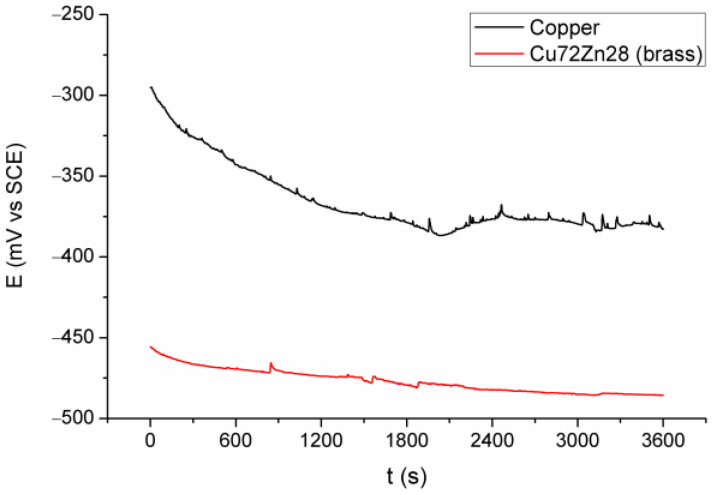
OCP for Cu and brass (Cu72Zn28) in fermented fruit juice at 25 °C for 3.6 ks.

**Figure 3:**
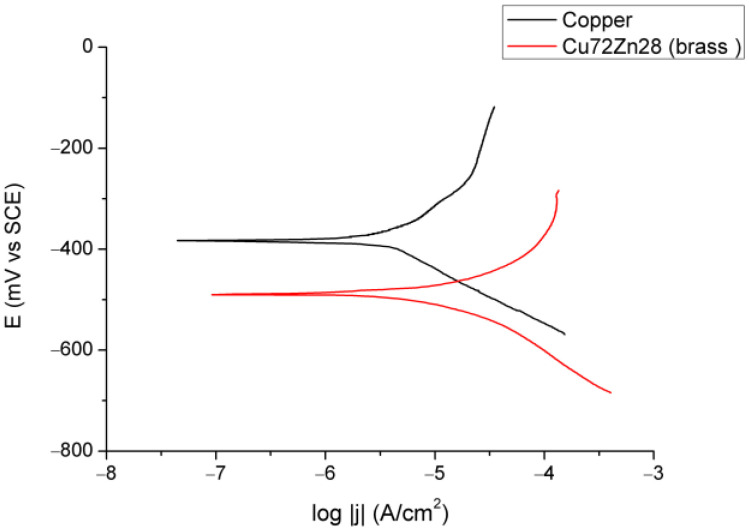
Tafel plots for Cu and Cu72Zn28 in fermented fruit juice at 25 °C and scan rate of 10 mV/min.

Figure 2 and Figure 3 show the results of electrochemical experiments; Figure 2 shows OCP for copper and brass (Cu72Zn28) in fermented fruit juice, Figure 3 shows polarization curves in the apparent Tafel’s region for copper and Cu72Zn28 alloy in the same corrosion environment.

A similar appearance of the curves for Cu and Cu72Zn28 is shown in Figure 2; for both of them, OCP generally decreases with time, and the curves are rough, especially the Cu curve, with many small and sharp peaks and troughs. The curve for the Cu had a small rise after about two thousand seconds before stabilization. Figure 2 also shows that OCP for the Cu electrode is higher for 80–160 mV during the measurement and approximately 100 mV at the end of the measurement.

In Figure 3, the polarization curves for the Cu and Cu-Zn alloys in various electrolytes are almost commonly shaped, with narrow Tafel areas that had the linear part (in the logarithmic diagram) of about 40–50 mV (starting at overpotentials of approximately 50 mV to near 100 mV) in the anodic direction. Table 1 and Table 2 show the results of the Tafel extrapolation method, which are analyzed later in the text.

In Table 1, *β_A_* and *β_C_* are anodic and cathodic Tafel slopes, respectively, which were obtained experimentally from the Tafel extrapolation method.

The Stern–Geary coefficient *B* (with a voltage unit) is calculated using the equation:

(1) $$B=\frac{\beta_{A}\cdot |\beta_{C}|}{2.3\cdot (\beta_{A}+|\beta_{C}|)}$$

which is used for the calculation of Icorr after experimental determination of *R_P_* by the LPR method, by:

(2) $$B=I_{CORR.}\cdot R_{P}$$

Both the above formulas are part of the well-known Stern–Geary equation [41]. The symbols explained above are used in Table 2. *E_CORR_*_._ is an experimental value separately obtained from the two methods given in the first raw or the table. These values should be identical to or very close to the OCP values (Table 1), which are obtained significantly well.

The data in Table 1 and Table 2 show that both materials have high corrosion resistance in the environment. Copper in its pure form has a 4.1-times-higher polarization resistance and 3.77- to 3.80-times (depending on the used method) lower corrosion current density, which is significantly better performance. OCP for Cu is also more positive for about 100 mV, suggesting a lower corrosion rate in the tested solution. LPR has marginally higher values for corrosion current density than Tafel, but the consistency of the results between the two methods used was more than convincing. In absolute values, the Cu corrosion rate is approximately 0.027 mm/year or 37 years for a mm of corroded metal. This confirms that, under the tested conditions, it is a very suitable material for the purpose, but it is not so for brass with 0.102 mm/year (needing nearly 10 years to cause 1 mm of uniform corrosion). The practical implications of these findings are discussed in the last paragraph of this paper.

Michels, H.T. et al. (2005) [42] reported that copper surfaces exhibited a 99.9% reduction in microbial activity, whereas brass demonstrated a significantly weaker antimicrobial effect. The study highlights that copper reduces MIC risk by inhibiting microbial biofilm formation, thereby supporting the conclusion that copper outperforms brass in resisting microbiologically influenced corrosion.

**Figure 4:**
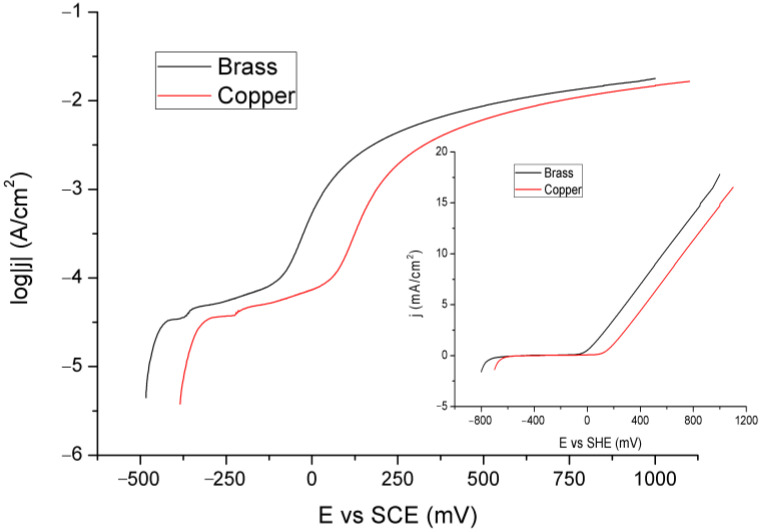
LSV curves for the Cu and brass electrodes.

Figure 4 presents the LSV curves for the Cu and brass electrodes.

The linear graph, presented in the inset of Figure 4, shows a monotonic increase in current density with potential for both of them. There are no prominent features, such as peaks, on the curves. To draw better conclusions, the logarithmic dependence of current on potential is given in the main (larger) part of this figure. In it, there are two shoulders; the first is more pronounced for Cu than brass, and the second is broader but less flat (the current increases more slowly with potential in the area). In this way, a particular and rapid electrochemical reaction is not emphasized, but it could indicate the formation of a corrosion film. Since they are almost identical, they are most likely related to Cu and not zinc reactions, which probably only pass into solution from even lower potentials by oxidation to Zn^2+^ ions. The first shoulder is in the area of potential that could be attributed to the formation of copper oxide, and the second is possibly in correlation with the formation of carbonates (hydroxycarbonates). The rough (jagged) parts of the curves (between the shoulders and just above them) indicate film formation and dissolution reactions.

Thermodynamic calculations were performed for the final product of the production process—fermented apricot juice. The precise control and measurement of some parameters during fermentation are beyond the scope of this study. Even the studies where fermentation was one of the main topics sometimes performed just the concentrations of the main ingredients at the finish of the process [43] or monitoring of just a few basic parameters like pH, total acidity (by titration), soluble solids content, and total sugars [44] and use modern analytics (for organic components) just for the final product of fermentation. Those studies used commercial yeast monocultures similar to those in this paper (musts were inoculated with commercial yeast). This paper shares the same methodology with them in the way that fermentation is partially, although minorly, carried out in a spontaneous manner. This is even more significant here since the traditional method of producing distillate from apricot must have been followed.

**Figure 5:**
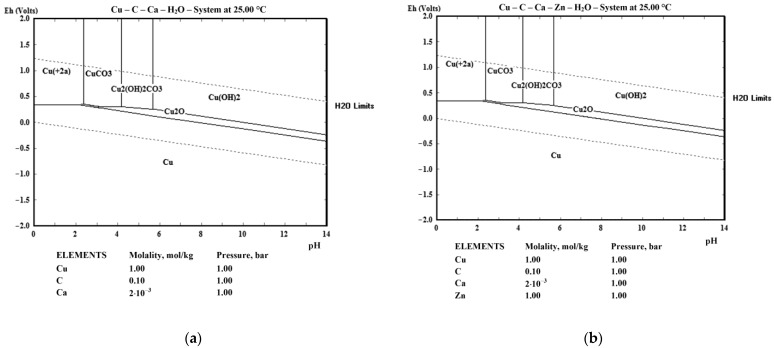
E-pH diagrams for: (**a**) Cu and (**b**) brass in 0.1M acetic acid and 2.0 mmol Ca.

E-pH diagrams constructed for Cu and brass in 0.1M acetic acid (generally the total concentration of all organic acids) with calcium at a concentration of 2.0 mmol/L (80 mg/L) are shown in Figure 5. Previous concentrations are rounded from the actual concentrations in fermented fruit juice of 0.09 M of acetic acid and 75.8 mg/L (ICP analysis) for the calcium to present E-pH diagrams as typical. Both approximations do not affect the E-pH diagram but could be easily expanded in a multi-concentration E-pH diagram, which was the main reason for the procedure. It should be emphasized here that there is naturally a mixture of organic acids in the fermented juice (e.g., malic and citric, which were from the fruit and were not derived from a fermentation). Still, all of them are in small proportions compared to acetic acid. For example, for the proportion of lactic acid to be even slightly significant, a large proportion (or monoculture) of specific strains of bacteria, such as Lactobacillus spp. or Bifidobacterium spp., is required [45]. The same applies to the calcium content since not all of it originated from the fruit but also from added water. The fermented juice contained nearly 10% of the dwell water, which was hard (15.6 °dH, 279 mg/L CaCO_3_ eq.) and contained 94.3 mg/L of calcium. Figure 5 was drawn in HSC Software (Version 6.12) [46].

**Figure 6:**
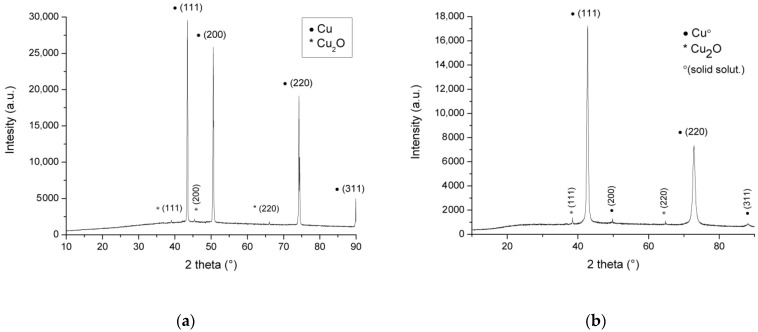
XRD diagram for oxidized (by LSV) samples: (**a**) Cu, (**b**) brass.

It should be highlighted that apricot juice contains potassium and magnesium in concentrations comparable to that of calcium. Nevertheless, both metals, with concentrations in the range of millimoles, do not affect E-pH diagrams and were detected by EDS analysis in corrosion films in very low concentrations. Thus, they were not included in the calculation and were not determined by chemical analysis. It can be concluded that the following compounds can be expected in the anode films: Cu_2_O, CuCO_3_, and Cu(OH)_2_CO_3_. Nevertheless, after investigation of the XRD results of the oxidized samples after the imposed LSV pulse (Figure 6), analysis strongly indicates that the only possibility of the corrosion product is Cu_2_O, which can be explained with the long strip of Cu_2_O stability from a strong alkaline solution (pH = 14) till acid environment up to pH = 2.12 (2.35, upper line). It is highly narrow between pH = 2.1 and 3.5, which is very near the starting pH value of the electrolyte. It should also be considered that Cu^2+^ is stable above +337 mV vs. standard hydrogen electrode (SHE) potential and pH lower than 2.12, CuCO_3_ in the ranges of 2.35 < pH < 4.16 and more positive potential of 299 mV SHE and Cu_2_(OH)_2_CO_3_ while 4.16 < pH < 5.68 and potentials above 254 mV (the highest pH) and 299 mV (the lowest pH in the stated range).

During apricot juice fermentation, organic acids (e.g., lactic, acetic) produced primarily by bacterial strains create an acidic environment. This pH drops, driven by microbial metabolites [47,48] and this can accelerate corrosion in fermentation equipment. Such microbial activity may explain the observed pH range of 2.1–3.5 in E-pH (Pourbaix) diagrams, which governs the stability of dissolved Cu^2^⁺ ions and the formation of corrosion products like Cu_2_O or Cu(OH)_2_ [49]. Because of this, Cu_2_O is only an obligatory/expected species, even with a broader area of stability of CuCO_3_ regarding potential range. The reaction with lower electrode potential will continue to higher potentials, so CuCO_3_ is not necessarily to be obtained even if the stability area covers the largest part of the LSV experiment. Additionally, local pH could vary in both directions, and Cu^2+^ and Cu hydroxide carbonate could also result from the LSV experiment.

Figure 6 shows XRD patterns of electrodes after LSV treatment. The 2θ in the XRD figures is the angle between the transmitted beam and reflected beam.

In Figure 6, Cu and copper solid solution patterns are only clearly visible on diagrams with barely noticeable (200) and (311) in the copper solid solution diffractogram. The diffraction pattern for the Cu sample shows peaks for Cu at 43.4, 50.5, 74.2, and 89.9 degrees 2θ. The peaks for brass are just to the left of these (42.7°, 49.7°, 72.8°, and 88.2°), as expected [50]. XRD peaks at 39.02°, 45.40°, and 66.08° diffracted from the (111), (200), and (220) planes of the fcc structure Cu_2_O, and they are right from peaks for the pure cuprite mineral, similar to organically obtained nano-particles of Cu_2_O [51]. The intensities of the Cu_2_O peaks were low, which suggests very thin oxide films at the surface of the samples (<1 μm), similar to the findings that the intensity of XRD peaks Cu metal films as a function of the film thickness [52]. The (200) plane for Cu is almost missing in Figure 6b due to the low intensity of that peak (at 49.7°) of the brass. Peaks with low intensity for Cu(I) oxide also indicate a high porosity of the corrosion layer and probably low surface coverage with a high degree of the free metal (alloy) surface, which is additionally supported by the high intensities of the Cu and the solid solution of Cu in Figure 6a,b. Based on the discussion above, it can be assumed that the (possibly larger) part of the copper oxide is in amorphous form.

**Figure 7:**
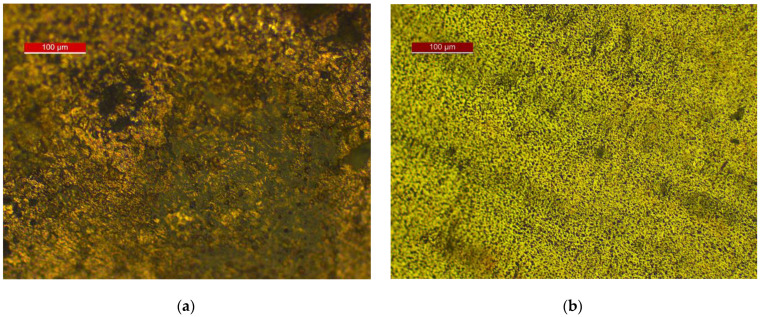
Reflected light optical microscopy of corrosion films after LSV treatment at surfaces of: (**a**) Cu; (**b**) brass.

**Figure 8:**
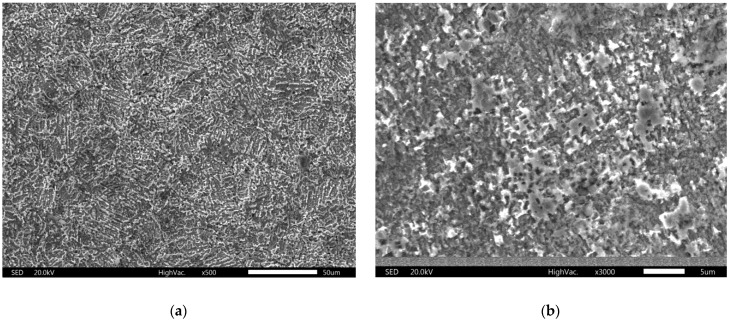
SEM images of corrosion film after applying the LSV method: (**a**) Cu; (**b**) brass.

**Figure 9:**
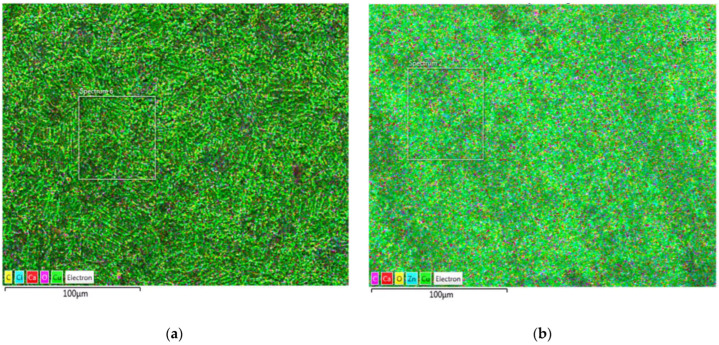
EDS images with an element distribution map of the corrosion film after LSV: (**a**) Cu; (**b**) brass (C, Cl, Ca, O, Cu for both, plus Zn on the right for brass).

Figure 7, Figure 8 and Figure 9 show the results of optical and scanning electron microscopy. Table 3 shows the results of the EDS analysis (Cu corrosion film—Spectrum 6 on Figure 9a and Cu72Zn28 corrosion film—Spectrum 2 on Figure 9b).

The optical images show a high coverage of the surface of the electrodes (Figure 7) with corrosion products. Optical microscopy exclusively provides information on the color of the surface, which is not the case with SEM imaging. That insight shows the Cu_2_O (in dark brown or black parts of the surface), but it also indicates the possibility of a mixture with carbonate on the copper specimen (greenish parts of Figure 7a); certainly, hydroxycarbonate and/or carbonate (green parts) for brass and a high probability of the Cu_2_O existence (dark areas on the surface) on brass (Figure 7b).

The data in Table 3 (EDS analysis of the average composition of the surfaces in Figure 9) suggest the possibility of Cu(I) oxide existence on the surface. The ratio of Cu to oxygen is approximate, as in the compound, although this can only be taken as circumstantial evidence. However, in Figure 9, where the surface is mapped (where the analyses are from), it can be seen that the entire surface is evenly covered with a corrosion film. It is even more apparent that Cu_2_O covers the whole surface in the SEM images (Figure 8). The corrosion film appears homogeneous in the micrographs. There are no signs of pitting corrosion in these images. Figure 9 (EDS images with an element distribution map) further confirms this homogeneity and suggests a flat surface [53,54]. However, it allows the corrosion layer to be porous and thin (microsized), which was concluded by XRD analysis. It also (additionally) suggests the amorphous nature of the corrosion products [54], which could explain weak peaks of Cu_2_O in the X-ray diffractogram.

**Figure 10:**
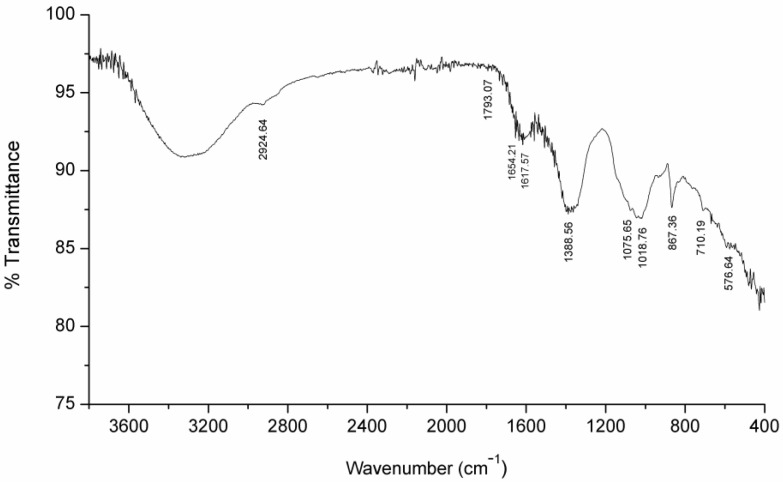
FTIR spectrum of corroded copper sample.

Finally, the relatively high calcium content in the corrosion film (relative to the liquid) hints at the tantalizing idea that it is due to (anaerobic) bacteria that precipitate calcium (in corrosion processes influenced by methanogens), especially with consideration that it was proven in another study [55] that Ca was present in the corrosion layer of the coupon incubated with M. maripaludis Mic1c10. This hypothesis is particularly intriguing because CaCO_3_ precipitation at low pH, such as in the tested medium, is highly unlikely (even with local pH change at the electrode). FTIR spectroscopy analysis was used with the aim to resolve the issue of carbonate presence, and the spectrum is shown in Figure 10.

The major bands identified for CaCO_3_ in the literature were found at 577, 713, 856, 873, 1021, 1082, 1456, 1794, 2515, 2872, 2925, and 3450 cm^−1^ [56]. The results in Figure 10 are in fair agreement with the typical CaCO_3_ spectrum, with bands found at 577, 710, 867, 1019, 1076, 1389, 1793 and 2925 cm^−1^. However, only a sharp and intense band is at 867 cm^−1^, and to some extent, a band at 1389 cm^−1^. All other bands are very weak; at 710, 1019, and 2925 cm^−1^ were shoulder bands. Two peaks (at 1619 and 1654 cm^−1^) are not characteristic IR bands for CaCO_3_. The results of the FTIR analysis are consistent with the authors’ assumption that the bands are like this due to the low concentration of CaCO_3_, which is additionally not evenly distributed over the surface of the sample, and that during analysis, it is difficult to hit the part of the sample where carbonates are dominant. Finally, these bands are probably overlapped with more intense peaks of components in higher concentration. Although the above FTIR results indicate the presence of CaCO_3_, it is not decisively proven here and the further research is necessary to confirm this possibility.

**Figure 11:**
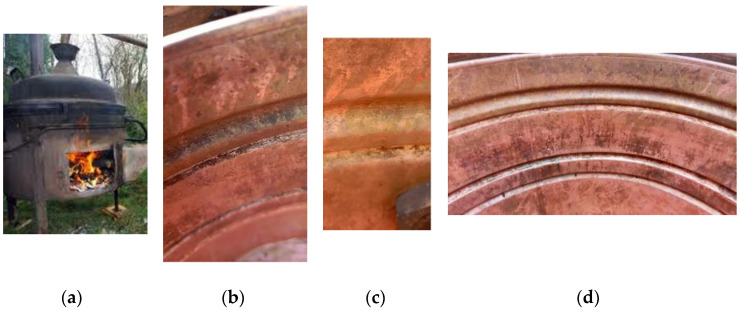
Copper kettle for distilling brandy: (**a**) kettle during the distillation process; (**b**–**d**) different sites of the copper kettle after the cleaning and drying (no polishing was performed).

Figure 11 shows a copper kettle for distilling brandy (a—external view, b–d—interior of the kettle after use).

Finally, but not by importance, the laboratory investigations’ results should be compared with authentic items in the application for which they are designed; in this case, the distillation of fermented fruit juice. During operation, the material is affected by MIC and general corrosion, and during standstill, atmospheric corrosion (in addition to washing and drying the equipment). Although the data show moderate corrosion in the working solution, it cannot be overemphasized that more significant corrosion processes occur on the surface (Figure 11a–d).

Figure 11a shows the batch distiller in operation (working volume of 100 L, temperature of the fermented liquid in the process 93–98 °C), and Figure 11b–d show the central part of the distiller (Cu cauldron), here from a smaller distiller (30 L) in Figure 11c (a slightly newer and with brighter surface). As can be seen in the pictures, some corrosion can be observed even after a thorough cleaning. The cauldron is dried and stored under room conditions. However, there are traces of a black corrosion film (most likely Cu(I) oxide) on many parts of the boiler, especially the grooves (of course, most of the surface is a shiny Cu surface). In one place in the groove, there is also a slight green coloration, which is probably Cu carbonate. This implies that the subject is complex and that further research is required to determine the particular impact of electrochemical, atmospheric, and microbiologically influenced corrosion.

To perform the quantitative analysis of the corrosion products in Figure 11a–d, scraped samples as powder were taken from the equipment, which was inaccessible to direct analysis. There was not enough sample for XRD analysis but EDS was performed and the typical composition of the powder was between the values given in Table 4.

The analysis in Table 4 shows that the powder sample consists of Cu_2_O and CuO. Spectrum 1 was closer to the cuprite composition and spectrum 2 was close to the CuO composition. Other spectra had Cu and O content between the spectrum 1 and 2 limits. The conclusion, drawn from the above results, was that these powders were a mixture of Cu oxides.

It is evident that pure Cu metal is suitable for the application, but the equipment requires good maintenance. Otherwise, it would be subjected to severe corrosion. Brass is less suitable for distillation even if the corrosion current is lower than 10 μA/cm^2^. Susceptibility to dealloying was not examined in detail, and hence, the acceptableness of the application needs further research. The lifespan of the Cu distiller equipment was not precisely determined in this study. Still, it is comparable with practical experiences that could last several decades and be maintained well. The higher temperature during distillation increases the corrosion rate, but on the other hand, equipment is used only several weeks per year, so the 2–3 mm of corrosion will indicate a lasting of about an entire century. This is in accordance with the industrial practice that the mean lifetime of a copper pot still (boil pot) is from 15 to 25 years [57] but with the upper limit of 25 years, which is limited by a decrease to half of its original thickness [58]. Considering the 240–250 working days per year of a middle-sized distillery [57], due to the seasonal use of 60 days yearly, the service life of small equipment in rural areas is quite analogous to 60–100 years.

## 4. Conclusions

Several methods were used to characterize the corrosion properties of Cu and Zn in fermented apricot juice. It was determined that Cu has a nearly four-times-lower corrosion rate in the investigated environment than zinc-based alloy. Tafel extrapolation and the LPR method had differences in results of less than 5%, which can be considered as an excellent agreement between them. Although there is a possibility that some Cu carbonate species can be part of the corrosion film, XRD did not confirm them. However, the SEM-EDS and optical microscopy indicate the possibility of carbonate species on Cu and brass electrodes. These methods confirm XRD findings for pure Cu metal. The thickness of the corrosion films already leads to weak peaks on XRD patterns even for Cu (I) oxide, demonstrating the difficulty of detecting any other corrosion products in the anodic film, especially if they are in amorphous form, which is highly possible for Cu carbonate and its hydroxide variation. Zinc carbonate could exist even in slightly acidic solutions (similar to the studied) but only at high electrode potentials. It is more stable in the form of the soluble ion (Zn^2+^), and Zn → Zn^2+^ + 2e^−^ is the main reaction of zinc from brass. Zinc oxide is stable only at pH above 6; its absence was unsurprising. At last, a combination of EH and atmospheric corrosion with MIC could lead to the severe manifestation of material decay (thick corrosion films), as demonstrated in this study. EDS and FTIR methods found strong indications of Ca and CaCO_3_ presence in corrosion film. Future research, which would include incubation with methanogens, must examine the presence of calcium in the corrosion layer in more detail to study the impact of MIC on the whole corrosion process in the system.

## Figures and Tables

**Table 1:** The Tafel slopes, coefficient of the Stern–Geary equation (*B*), and the OCP, fermented apricot juice, 25 °C (pH = 3.565).

Metal/Alloy	*β_A_*, mV/dec	*β_C_*, mV/dec	*B*, mV	OCP, mV vs. SCE, t = 3600 s
Cu	75.2	−72.6	16.04	−382.7
Cu72Zn28	62.5	−74.8	14.80	−485.6

**Table 2:** Corrosive parameters of a metal/alloy in fermented apricot juice, at 25 °C and a pH value of 3.565.

Metal/Alloy	Linear Polarization			Tafel	
	*R_P_*, kΩ·cm^2^	*I_CORR_*_._, μA·cm^−2^	*E_CORR_*_._, mV	*I_CORR_*_._, μA·cm^−2^	*E_CORR_*_._, mV
Cu	6.525	2.458	−383.4	2.350	−384.2
Cu72Zn28	1.596	9.273	−486.1	8.934	−488.3

**Table 3:** EDS analysis of corrosion products.

Element, wt%	C	O	Ca	Cu	Zn	Σ
Cu corrosion film	5.72	15.07	0.13	79.08	0.00	100.00
Cu72Zn28 corrosion film	3.34	12.97	0.14	61.00	22.55	100.00

**Table 4:** EDS analysis of scraped (black colored) corrosion products from the equipment in Figure 11.

Element, Atomic %	O	Cu	Σ
Spectrum 1	36.41	63.59	100.00
Spectrum 2	45.24	54.76	100.00

## Data Availability

The original contributions presented in the study are included in the article, further inquiries can be directed to the corresponding author.
